# Identification of host genes leading to West Nile virus encephalitis in mice brain using RNA-seq analysis

**DOI:** 10.1038/srep26350

**Published:** 2016-05-23

**Authors:** Mukesh Kumar, Mahdi Belcaid, Vivek R. Nerurkar

**Affiliations:** 1 Department of Tropical Medicine, Medical Microbiology and Pharmacology, Honolulu, USA; 2 Pacific Center for Emerging Infectious Diseases Research, John A. Burns School of Medicine, University of Hawaii at Manoa, Honolulu, Hawaii 96813, USA.

## Abstract

Differential host responses may be critical determinants of distinct pathologies of West Nile virus (WNV) NY99 (pathogenic) and WNV Eg101 (non-pathogenic) strains. We employed RNA-seq technology to analyze global differential gene expression in WNV-infected mice brain and to identify the host cellular factors leading to lethal encephalitis. We identified 1,400 and 278 transcripts, which were differentially expressed after WNV NY99 and WNV Eg101 infections, respectively, and 147 genes were common to infection with both the viruses. Genes that were up-regulated in infection with both the viruses were mainly associated with interferon signaling. Genes associated with inflammation and cell death/apoptosis were only expressed after WNV NY99 infection. We demonstrate that differences in the activation of key pattern recognition receptors resulted in the induction of unique innate immune profiles, which corresponded with the induction of interferon and inflammatory responses. Pathway analysis of differentially expressed genes indicated that after WNV NY99 infection, TREM-1 mediated activation of toll-like receptors leads to the high inflammatory response. In conclusion, we have identified both common and specific responses to WNV NY99 and WNV Eg101 infections as well as genes linked to potential resistance to infection that may be targets for therapeutics.

West Nile virus (WNV) is an enveloped positive-stranded RNA virus that has emerged over the last decade in North America to cause epidemics of meningitis, encephalitis, and acute flaccid paralysis in humans. While most human infections remain asymptomatic or develop a mild illness, a small subset (<1%) progress to severe neurological syndromes that include meningitis, acute flaccid paralysis, and encephalitis^1^. Although veterinary vaccines are available commercially, there is currently no approved vaccine or therapy for WNV infection in humans. Until 1999, WNV was geographically distributed in Africa, the Middle East, western and central Asia, India, and Europe, where it caused sporadic cases of febrile disease and occasional outbreaks of encephalitis in elderly people and in equines^2^. The unexpected emergence of WNV in the USA in 1999 was associated with the introduction of the NY99 strain, which is more virulent, and resulted in higher incidence of meningoencephalitis in humans as compared to the non-virulent strains such as the WNV Eg101 strain^3^. Recent outbreaks of highly virulent WNV strains have also been reported in the Mediterranean basin, southern Europe, and Russia^4^. The expansion of WNV disease globally highlights a need for greater understanding of mechanisms of immune control, including the cell-intrinsic processes that restrict infection.

WNV Eg101 was isolated in 1950 from normal appearing children near Cairo, Egypt. Seroprevalence among adults in the Nile Delta region has been demonstrated to be 61% to WNV Eg101 with little or no evidence of disease^5^. WNV Eg101 strain has 95.4% of nucleotide and 99.6% of amino acid identity to WNV NY99 strain. Both the WNV NY99 and WNV Eg101 strains are classified in same genotypic lineage and belong to same clade 1a of the lineage 1^3^. While the WNV NY99 strain is highly pathogenic and implicated in large-scale mortality, the WNV Eg101 strain is largely non-pathogenic^6^. We have previously demonstrated that virus-specific effector immune cell response limits virus replication and severe disease in mice infected with WNV Eg101 strain^7^. However, detailed mechanisms including the cell-intrinsic processes associated with differential pathogenesis of WNV NY99 and WNV Eg101 strains are largely unknown.

A better understanding of the global gene changes underlying the multi-step progression of pathogenicity during infection could help develop potential therapeutic strategies for emerging infectious diseases including WNV. Very recently, high-throughput RNA sequencing (RNA-seq) technology, which is a powerful way to profile the transcriptome with great efficiency and higher accuracy, has been employed in various viral infections and diseases^8^,^9^. RNA-seq technology is highly sensitive, quantitative, and provides unbiased measurement of gene transcripts in a large dynamic range of gene abundance. In this study, we used RNA-seq technology to analyze host transcriptomes in the brains of mice infected with WNV NY99 and WNV Eg101 to gain insight into the underlying mechanisms of the differential pathogenicity of these viruses. We have characterized and compared the host responses to the two WNV strains in terms of canonical pathways and biological functions, as well as in terms of individual genes.

## Results and Discussions

### Distinct and dynamic changes in the expression of cellular genes in WNV NY99- and WNV Eg101-infected mice brain

**Figure 1:**
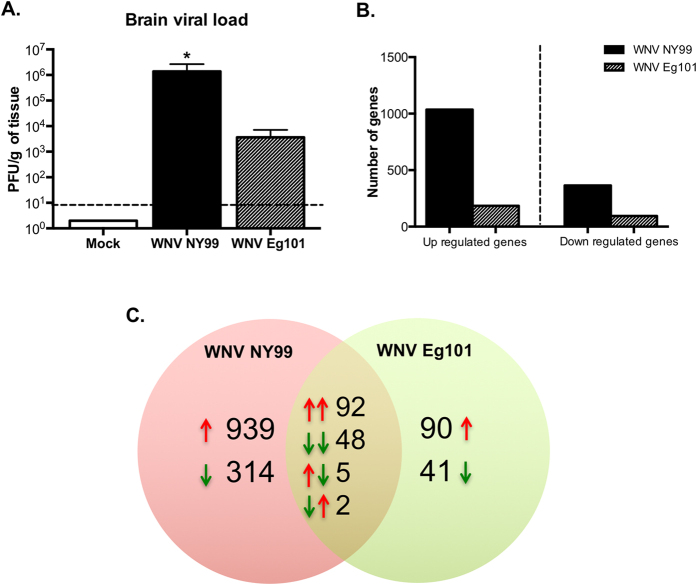
WNV infection of the brain causes changes in cellular gene expression. Mice were infected with 100 PFU of PBS (Mock), WNV NY99 or WNV Eg101 by footpad route. At day 8 after infection, brains (n = 4 per group) were harvested. (**A**) Brain viral load was determined by plaque assay using Vero cells and is reported as PFU per gram of tissue. Data represents the mean ± SEM, *P < 0.05. (**B**) Graph showing the number and fold change of up- and down-regulated genes. (**C**) A Venn diagram showing the number of differentially expressed genes in WNV NY99- and WNV Eg101-infected mice brains. Overlap comparison of differentially expressed genes detected in WNV NY99- and WNV Eg101-infected mice brains using the RNA-seq data set. Sets of up-regulated genes are represented by upward red arrows and sets of down-regulated genes are represented by downward green arrows. Pairs of arrows in the intersection refer to the direction of fold change in the comparisons on the left (WNV NY99) and right (WNV Eg101) hand sides.

To identify the dynamics of host gene expression associated with WNVE, RNA-seq analysis was conducted to explore the transcriptomes from mice brains infected with lethal, WNV NY99, and non-lethal, WNV Eg101 viruses, as well as mock-infected mice. After peripheral inoculation, WNV is first detected in the brain between days 4 and 6 after footpad inoculation and peak virus load is observed at day 8 after inoculation^10^,^11^. WNV NY99-infected mice demonstrate various neurological symptoms such as paresis, tremors, and ataxic gait at day 8 after inoculation. Therefore, to understand the transcriptome changes occurring at the time of high WNV replication in brain and when animals display clinical symptoms of encephalitis, we examined gene expression at day 8 after infection. Virus was not detected in the mock-infected brains. WNV was detected in the brains of both WNV NY99- and WNV Eg101-infected mice by plaque assay. However, virus titers were significantly higher in the brains of WNV NY99-infected mice when compared to WNV Eg101-infected mice (Fig. 1A). RNA harvested from the brains of mock-, WNV NY99- and WNV Eg101-infected mice (four mice per group) were subjected to high-throughput sequencing on an Illumina HiSeq 2500 sequencer as described previously^8^,^9^.

Gene expression profiles for WNV NY99- and WNV Eg101-infected brains were compared with mock-infected brains. Figure 1B demonstrates total numbers of up- and down-regulated differentially expressed genes for WNV NY99- and WNV Eg101-infected mice. Number of differentially expressed genes was higher in the brains of WNV NY99-infected mice, which correlate with significantly higher brain viral load observed in these mice compared to WNV Eg101-infected mice (Fig. 1A). In WNV NY99-infected mice, 1,036 genes were up-regulated with log_2_ fold change values ranging from 0.48 to 9.6 and 364 genes were down-regulated with log_2_ fold change values ranging from −0.48 to −9.6. In WNV Eg101-infected mice, 184 genes were up-regulated with log_2_ fold change values ranging from 0.48 to 7.4 and 94 genes were down-regulated with log_2_ fold change values ranging from −0.48 to −2.6 (Fig. 1B). The number of up-regulated genes was larger than the number of down-regulated genes in both groups of virus-infected mice. Genes involved in biological pathways related to innate antiviral immunity and inflammation responses were among the top differentially expressed genes after WNV NY99 infection. These include well-characterized responses such as type I interferon (IFN) signaling and chemokine signaling. Similar to WNV NY99, genes associated with IFN signaling were among the top differentially expressed genes after WNV Eg101 infection. Tables 1 and 2 depict the top 10 up-regulated and down-regulated genes in WNV NY99- and WNV Eg101-infected mice brains, respectively. The complete list of differentially expressed genes can be found in Supplementary tables S1 and S2.

Furthermore, Venn diagram was generated to examine the unique and overlapping genes for both groups. As depicted in overlapping circle in Fig. 1C, 147 genes were common to infection with WNV NY99 and WNV Eg101. Nighty two up-regulated genes and 48 down-regulated genes were common to infection with both viruses, indicating that 50% (92/184) of genes that are up-regulated in the brain following infection with WNV Eg101 are also up-regulated following infection with WNV NY99 and that 51% (48/94) of genes that are down-regulated in the brain following infection with WNV Eg101 are also down-regulated following infection with WNV NY99. Interestingly, seven genes common to infection with both the viruses demonstrated inverse trend (Table 3). Expression of HOXB5, GFAP, HBA-A1, CYR61 and α2M genes was up-regulated in WNV NY99 infection and down-regulated in WNV Eg101 infection. In contrast, expression of SYNPO2 and PTPN3 genes was down-regulated in WNV NY99 infection and up-regulated in WNV Eg101 infection. GFAP and α2M genes are particularly of interest. GFAP is an astrocyte marker and its up-regulation has been demonstrated in WNV infection. Others and we have demonstrated that activation of astrocytes is associated with enhanced production of pro-inflammatory mediators after WNV infection^12^,^13^. α2M is a protease inhibitor and cytokine transporter with important role in inflammation. α2M is synthesized in the brain primarily by astrocytes, and expression of its receptor has been identified in neurons and astrocytes^14^. Several studies demonstrated that α2M is a specific cytokine carrier, which binds major pro- and anti-inflammatory cytokines, such as interleukin (IL)-1β, IL-6, TNF-α, and others^15^. It has been demonstrated that α2M^−/−^ mice are resistant to the lethal effects of lipopolysaccharides^16^. α2M is also implicated in Alzheimer disease (AD) due to its ability to mediate the clearance and degradation of α-beta, the major component of beta-amyloid deposits^17^. However, role of α2M is yet to be determined in WNV infection and other inflammatory infectious diseases. Up-regulation of both GFAP and α2M genes in brain cells especially astrocytes may contribute to enhanced inflammation observed in the brains of WNV NY99-infected mice.

Genes that were up-regulated in infection with both the viruses are mainly associated with IFN signaling. Interferon stimulated genes (ISG) such as MX1, IFIT1, IFIT2, IFIT3, and RSAD2 (viperin) were significantly up-regulated after infection with both the viruses (Table 4). The IFN response is central to the innate defense mechanisms of the host against WNV infection^18^. The paracrine and autocrine secretion of IFN renders cells “antiviral” by inducing several genes including ISG, the dsRNA-dependent protein kinase R (PKR) and 2′-5′-oligoadenylate synthase (OAS)/RNase L. These ISG confer an antiviral state by blocking virus replication at different levels such as early-stage virus infection, inhibition of post-transcriptional modification and virus maturation^18^. It has been demonstrated that viperin can inhibit WNV infection in cells and *in vivo*^19^. Members of the IFN-inducible tetratricopeptide (IFIT) family of genes (IFIT1, IFIT2, and IFIT3) also contribute to the control of WNV infection^18^. IFIT2 is important for controlling virus replication in the brain^20^. The absence of PKR signaling or a deficient OAS/RNaseL pathway also leads to increased susceptibility to WNV infection^21^,^22^. Genetic variation in OAS1 also has been suggested as a risk factor for initial infection with WNV in humans^23^.

Interestingly, genes associated with inflammation such as cytokines, chemokines and their receptors were exclusively expressed only after WNV NY99 infection. WNV NY99 infection induced a strong up-regulation of multiple cytokines and chemokines in mice brains (Table 4). Chemokine pathway-associated genes, such as C-X-C motif chemokine 10 (CXCL10), chemokine (C-C motif) ligand 5 (CCL5), and CCL2, were among the 10 most up-regulated genes after WNV NY99 infection. CXCL10 was the topmost up-regulated gene in WNV NY99-infected mice. Most of the chemokine, cytokines and their receptors were significantly up-regulated after WNV NY99 infection, including CXCL1, CXCL13, CXCL16, CCL7, CCL4, CCL3, CCL8, CCL19, IL-1β, IL-1α, and IL-7. In contrast, WNV Eg101 infection did not induce the expression of these pro-inflammatory mediators (Table 4). Only CXCL12 gene was up-regulated in WNV Eg101 infection. Similar to IFN response, WNV-induced pro-inflammatory mediators are also known to protect mice from lethal WNV disease. CXCL10 promotes trafficking of WNV-specific CD8^+^ T cells via binding to its cognate receptor CXCR3^24^. Enhanced expression of CCL3, CCL4, and CCL5 by WNV infection leads to CCR5-dependent trafficking of CD4^+^ and CD8^+^ T cells, NK cells, and macrophages into the brain^25^. CCL2 is important in the trafficking of inflammatory monocyte into the brain after WNV infection^26^. IL-1β promotes immune cell trafficking into the brain^27^. Interestingly, we observed an enhanced production of key pro-inflammatory cytokines and chemokines in only WNV NY99-infected mice despite increased disease severity observed in these mice. This could be due to significantly increased antigen load in WNV NY99-infected mice compared to WNV Eg101-infected mice (Fig. 1A).

Collectively, we demonstrate both predicted and novel gene changes in response to infection with lethal, WNV NY99 and non-lethal, WNV Eg101 viruses. Previous studies have not reported the entire transcriptome profile of WNV-infected mice brain using RNA-seq. Qian and colleagues reported RNA-seq analysis of WNV-infected human macrophages *in vitro*^28^. Similar to our data, this study also identified cytokine/chemokine signaling and IFN signaling as key signaling pathways modulated by WNV infection in human macrophages. Further, Qian and colleagues demonstrated the important roles of IFI27, AIM2, CCR3 (receptor of chemokines CCL5 and CCL8) and CXCR3 (receptor of chemokines CXCL10) in resistance to infection with WNV. Our data also demonstrates up-regulation of CCL5, CCL8, and CXCL10 in WNV NY99-infected mice (Table 4). However, we did not observe up-regulation of IFI27 and AIM2. This could be due to the differential antiviral response to WNV infection in peripheral macrophages compared to resident brain cells.

The major set of genes/pathways with increased expression observed in the present study with WNV are similar to those reported for other neuroinvasive viruses like Japanese encephalitis virus (JEV). Clarke and colleagues conducted microarray analysis of WNV NY99- and JEV-infected mice brains and reported that flaviviruses-induced transcriptional changes in the brain include the differential expression of genes associated with inflammation, apoptosis, interleukin 17 receptor A, IFN signaling, glutamate signaling, and tRNA charging pathway^29^. Similarly, our data also demonstrate induction of aforementioned genes in the brain after WNV NY99 infection; however, we did not observe the differential expression of genes associated with tRNA charging pathway. This could be due to the difference in the mice strain (C57BL/6J vs. Swiss Webster) and route of inoculation (subcutaneous vs. intracranial) used in the study by Clarke and colleagues. We inoculated mice by footpad route, which mimics the natural route of WNV infection. Other studies have also reported large-scale gene expression changes in the brain following subcutaneous JEV infection. Similar to our study, genes that are up-regulated in subcutaneous JEV infection are mainly associated with IFN signaling, pattern recognition receptors, chemotactic genes, activation of inflammasome, and cell death/survival signaling^30^,^31^. These results illustrate the pathways that are commonly activated during neurotropic viral infections of the central nervous system (CNS).

### Confirmation of RNA-seq data using qRT-PCR

**Figure 2:**
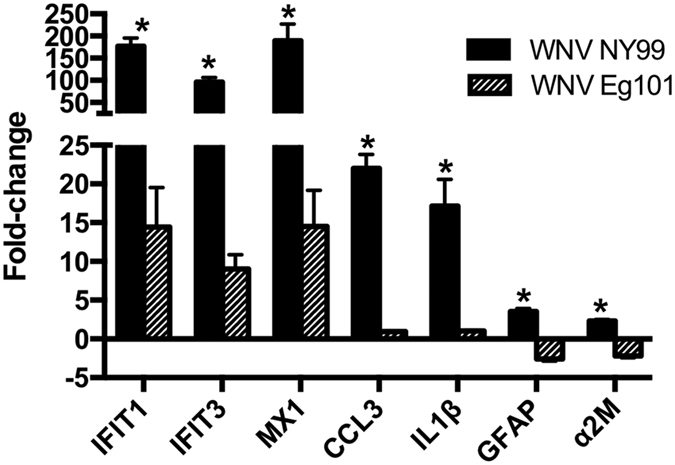
Validation of differentially expressed genes by qRT-PCR. qRT-PCR was conducted on RNA extracted from mock and WNV NY99- and WNV Eg101-infected mice brains at day 8 after infection to determine fold-change in MX1, IFIT1, IFIT3, CCL3, IL-1β, GFAP, and α2M gene expression. Changes in the levels of each gene were first normalized to the β-actin gene and then the fold-change in WNV-infected mice brain was calculated in comparison to corresponding mock-infected mice brain. Data represents the mean ± SEM, representing two independent experiments (n = 7 per group). *P < 0.05.

qRT-PCR was conducted on additional virus-infected (n = 7 in each group) and mock-infected (n = 4) animals to confirm the gene expression changes of a selected number of differentially expressed genes. Similar to RNA-seq data, MX1, IFIT1, and IFIT2 genes were significantly up-regulated in the brains of both WNV NY99 and WNV Eg101-infected mice compared to mock-infected mice (Fig. 2). The up-regulation of inflammatory mediators following WNV NY99 infection, but not following WNV Eg101 infection, was also confirmed by qRT-PCR. Pro-inflammatory mediators, IL-1β and CCL3, expression was only up-regulated in WNV NY99 infection. Others and we have previously demonstrated dramatic increase in the protein levels of multiple cytokines and chemokines in WNV NY99-infected mice brains at day 8 after inoculation, but this increase was not observed in the brains of WNV Eg101-infected mice^7^. Moreover, expression of GFAP and α2M genes was up-regulated in WNV NY99 infection and down-regulated after WNV Eg101 infection (Fig. 2).

### Functional analysis of WNV-modulated genes

**Figure 3:**
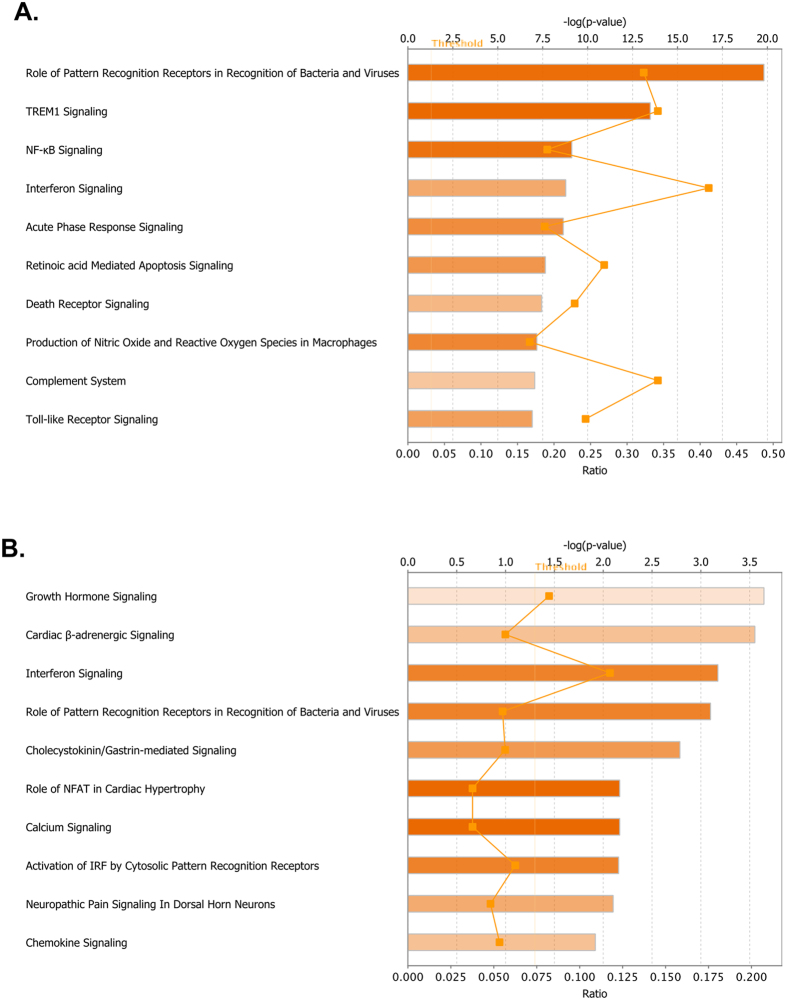
Top canonical signaling pathways activated by WNV NY99 and WNV Eg101 infections. Top ten canonical signaling pathways activated by (**A**) WNV NY99 and (**B**) WNV Eg101 infection in mice brains. Threshold bar indicates cut-off point of significance P < 0.05, using Fisher’s exact test. Line indicates ratio of genes in network to total number of genes in the canonical pathway.

To investigate the biological interactions of differentially expressed genes and to identify important functional networks, significantly modulated differentially expressed genes derived from RNA-seq analyses were imported into the Ingenuity Pathway Analysis (IPA) tool as described previously^8^,^9^,^32^,^33^,^34^. The highest activated networks (high z-score) were identified using IPA as described in the materials and methods. Figure 3A,B depict the top ten activated canonical pathways after WNV NY99 and WNV Eg101 infections, respectively. Consistent with the results from the analysis of individual genes, these pathways were associated with IFN, immune response and apoptosis signaling. The topmost activated canonical pathways after WNV NY99 infection included virus-sensing pathways such as ‘Pattern Recognition Receptors (PRR) in Recognition of Viruses and Bacteria’ and ‘Toll-like Receptor (TLR) Signaling’. In addition to virus-sensing pathways, key players in innate immunity and inflammation, such as ‘IFN Signaling’, ‘TREM-1 Signaling’, ‘Complement System’, and ‘Acute Phase Response Signaling’ were also highly activated (Fig. 3A). Moreover, pathological pathways such as ‘Death Receptor Signaling’ and ‘Retinoic acid-Mediated Apoptosis Signaling’ were also activated after WNV NY99 infection. Two of the top ten activated canonical pathways after WNV NY99 infection were shared with those affected by WNV Eg101 infection; ‘IFN Signaling’ and the ‘Role of PRR in Recognition of Bacteria and Viruses’ (Fig. 3B). Other highly activated pathways related to WNV Eg101 infection included ‘Growth Hormone Signaling’, ‘Cardiac β-adrenergic Signaling’, ‘Calcium Signaling’, and ‘Activation of IRF by Cytosolic Pattern Recognition Receptors’.

**Figure 4:**
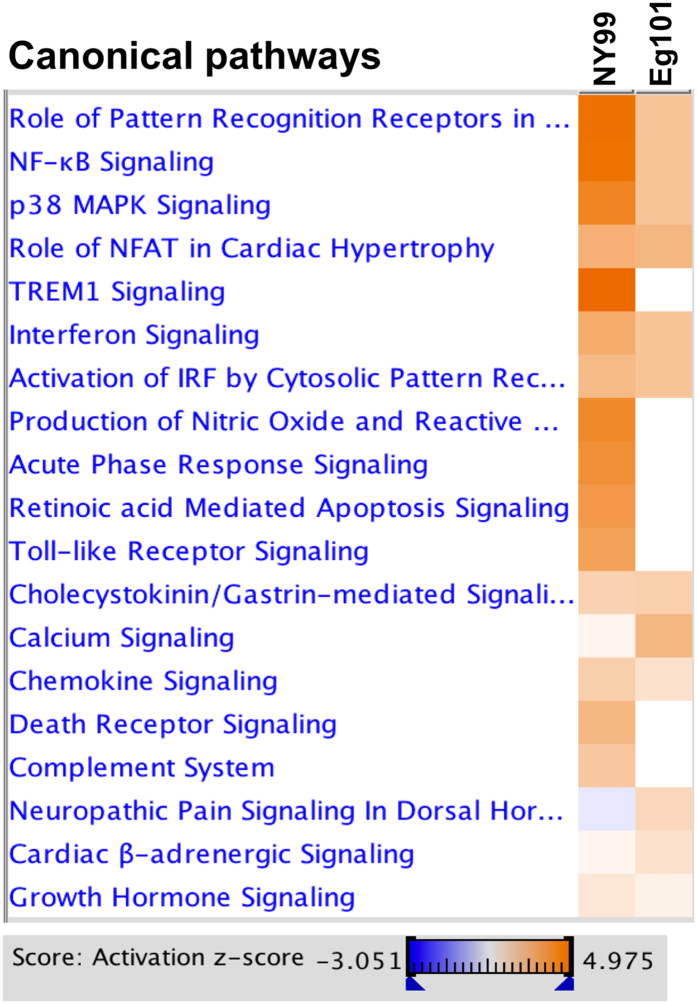
Comparison analysis of canonical signaling pathways activated by WNV NY99 and WNV Eg101 infections. Heat map showing top activated canonical pathways in WNV NY99 and WNV Eg101 infections. Networks shaded orange were up-regulated, while those in blue were down-regulated. Shading intensity indicates the degree that each gene was up-regulated or down-regulated. Range of activation z-score is also depicted in the figure.

We next generated heat map to compare the activated canonical pathways after WNV NY99 and WNV Eg101 infections. As depicted in Fig. 4, most of the canonical pathways were activated by infection with both the strains, although to different extents. Activation was more enhanced in WNV NY99-infected mice than in WNV Eg101-infected mice, which is consistent with higher brain viral titers observed in WNV NY99-infected mice. The three important canonical pathways activated exclusively in WNV NY99 infection were the ‘TREM-1 Signaling’, ‘Death Receptor Signaling’, and ‘TLR Signaling’. Pathways related to cell death were only activated after WNV NY99 infection. To further understand the role of these differentially activated canonical pathways after WNV NY99 and WNV Eg101 infection in disease pathogenesis, we generated the network maps of ‘PRR in Recognition of Viruses and Bacteria’, ‘TREM-1 Signaling’, and ‘Death Receptor Signaling’.

### Pattern recognition receptors

**Figure 5:**
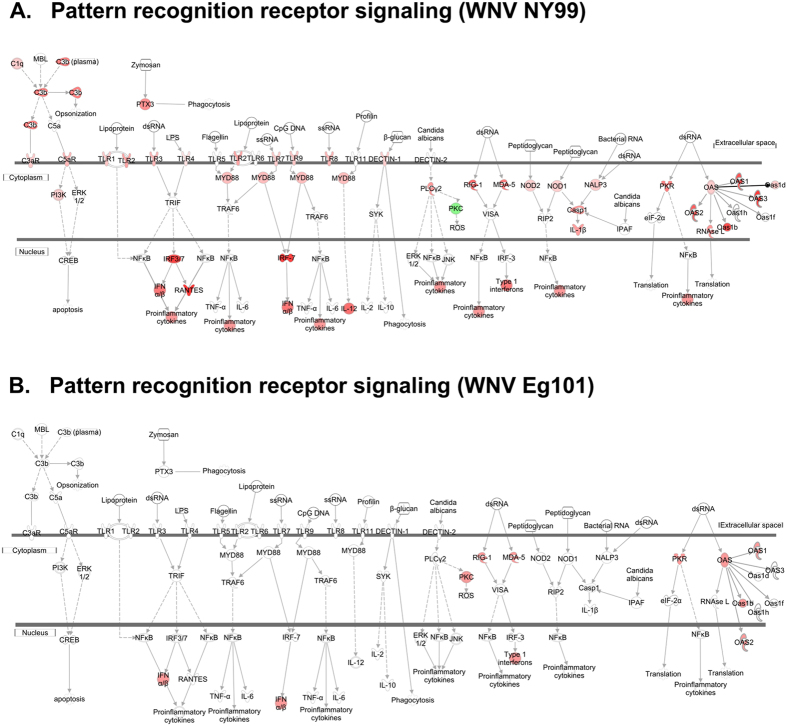
Pathway analysis for pattern recognition receptor signaling. Genes associated with pattern recognition receptors in virus sensing network activated by (**A**) WNV NY99 and (**B**) WNV Eg101 are shown. Differentially expressed genes are highlighted in color. Color intensity indicates the degree of up-regulation (red) or down-regulation (green) relative to the mock-infected mice brains. Solid lines represent direct interactions and dashed lines indirect interactions.

The innate immune system encodes a series of PRR that, upon recognition of a viral pathogen-associated molecular pattern (PAMP), induce a potent antiviral host immune response^35^. The activation of these PRR is very tightly regulated at multiple steps to prevent tissue damage due to uncontrolled production of cytokines. The major PRR are the retinoic-acid inducible gene-I (RIG-I)-like receptors (RLR), TLR, and the nucleotide oligomerization domain (Nod)-like receptors (NLR). Members of the RLR (RIG-I and MDA5) and TLR (TLR3 and TLR7) families are the dominant PRR that detect WNV infection^36^,^37^. These receptors drive complementary antiviral defense programs by activation of downstream signaling resulting in the production of antiviral IFN, ISG and pro-inflammatory cytokines^36^,^37^. Our data demonstrate that WNV NY99 infection induces the expression of all three major PRR; RLR, NLR and TLR (Fig. 5A). Interestingly only RLR were induced after WNV Eg101 infection (Fig. 5B). The RLR are a family of cytosolic RNA helicase proteins comprised of three members: RIG-I, myeloma differentiation antigen 5 (MDA5), and LGP2^36^. The importance of the RLR signaling pathway in protection against WNV has been demonstrated by several studies *in vivo*. RLRs detect and respond to WNV by inducing a potent antiviral defense program, characterized by production of type I IFN and expression of antiviral effector genes including ISG. Both RIG-I and MDA5 are required for optimal antiviral responses against WNV^38^,^39^. RIG-I or MDA5 knockout mice show increased mortality compared to wild type mice after WNV infection^40^,^41^.

### Toll-like receptors

While RLR pathway is known to be protective after WNV infection, the role of TLR pathway has been controversial. The TLR family is composed of more than 10 members, with each acting as a sensor of conserved microbial component, that drive the induction of immune responses^42^. Recognition of WNV by TLR occurs in endosomes and is largely mediated by TLR7 and TLR3, which bind ssRNA and dsRNA, respectively. The role of TLR3 in WNV infection remains controversial, as two independent studies using the same mouse model demonstrated opposing phenotypes of enhanced susceptibility and resistance to WNV infection^43^,^44^. Similarly, the function of TLR7 in WNV infection is less clear, as one study found no difference in susceptibility to WNV between wild-type (WT) and TLR7^−/−^ mice after intradermal WNV infection^45^, and in other study TLR7^−/−^ mice were more susceptible to intraperitoneal WNV infection than WT mice^46^. Protection in TLR3 knockout mice is attributed to the reduced production of pro-inflammatory mediators from brain cells, which in turn reduce primary neuronal damage and promote blood-brain barrier stability^43^. Knockout of both TLR3 and TLR7 have little systemic impact on IFN production after WNV infection. Our data demonstrate that WNV NY99 infection induces the expression of seven members of TLR family including TLR1, TLR2, TLR3, TLR4, TLR7, TLR8, and TLR9 (Fig. 5A and Table 5). In contrast, WNV Eg101 infection does not induce expression of any TLR (Fig. 5B and Table 5). Furthermore, we observed increased levels of several pro-inflammatory mediators and their cognate receptors only in the brains of WNV NY99-infected mice, which correlate with TLR activation in these mice.

Although TLR are important for viral recognition, excessive TLR activation may result in a pathogenic rather than a protective outcome in infections of the CNS. In this regard, antagonizing TLR signaling has been suggested as a therapeutic approach to control brain inflammation and overall disease pathology^43^,^47^,^48^. Other than WNVE, activation of TLR pathway is also demonstrated to be associated with encephalitis in the SIV model of neuroAIDS^49^. Recent study demonstrated that macrophages from young individuals can down-regulate TLR3 following infection with WNV, whereas macrophages of the elderly cannot, resulting in production of high levels of pro-inflammatory and vasculogenic cytokines in this group^50^. This is an interesting observation as elderly individuals are far more susceptible to develop WNVE than young individuals^51^. Moreover, TLR4 signaling during infection with highly pathogenic H5N1 influenza A virus has been reported to contribute towards lung pathology^52^. Similar to our study, it has been recently demonstrated that peripheral inoculation of WNV Eg101 in WT mice at doses lower than 10^6^ PFU did not produce any clinical sign of disease or mortality, and no TLR activation^53^. However, inoculation of WNV Eg101 at a higher dose (10^7^ PFU) induced significant mortality and TLR activation, suggesting that activation of TLR pathway is a result of high virus replication^53^. Similarly, in this study, high virus load in WNV NY99-infected mice results in TLR activation and subsequently enhanced inflammation observed in these mice.

### Nod-like receptors

**Figure 6:**
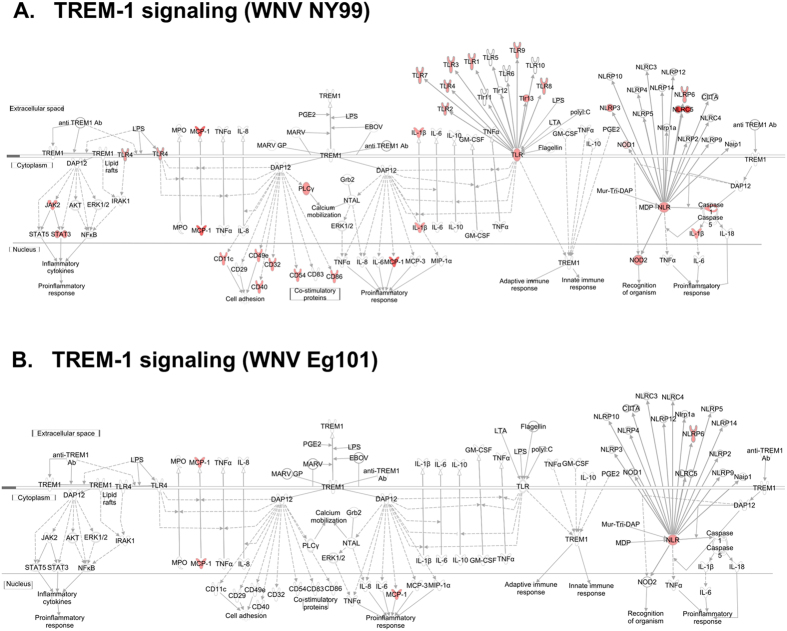
Pathway analysis for TREM-1 signaling. Genes associated with TREM-1 signaling activated by (**A**) WNV NY99 and (**B**) WNV Eg101 are shown. Differentially expressed genes are highlighted in color. Color intensity indicates the degree of up-regulation (red) or down-regulation (green) relative to the mock-infected mice brains. Solid lines represent direct interactions and dashed lines indirect interactions.

NLR are soluble or cytosolic receptors in the mammalian cell cytoplasm. Our data demonstrate activation of NOD1, NOD2, NLRP3, NLRP6, and NLRC5 following WNV NY99 infection (Figs 5A and 6A). In contrast, only NLRP6 was induced after WNV Eg101 infection (Figs 5B and 6B). NLRC5, and NLRP3 can form inflammasomes by recruiting apoptosis-associated speck-like protein containing a CARD (ASC) to activate caspase-1, leading to the cleavage of pro-IL-1β to mature IL-1β. Activation of the NLRP3 inflammasome and subsequent secretion of IL-1β family pro-inflammatory cytokines have been implicated in the protection against WNV infection^54^. NLRP3 inflammasome has also been demonstrated to play role in the pathogenesis of Japanese encephalitis^55^. We have previously demonstrated that ASC is critical for the immune response and survival in WNVE^56^. However, role of NLRC5 and NLRP6 is yet to be determined in WNV pathogenesis. In addition to RLR, TLR, and NLR, PKR and OAS are classes of IFN-inducible PRR that can recognize dsRNA and restrict a number of viruses, including WNV. Our data demonstrate activation of PKR and OAS after infection with both the viruses (Fig. 5A,B).

Collectively, this analysis demonstrated that differences in the activation of key PRR resulted in the induction of unique innate immune profiles, which corresponded with the induction of IFN and inflammatory responses.

### TREM-1 signaling

TREM1 is known to activate neutrophils and monocytes/macrophages by signaling through the 12-kDa-adapter protein DNAX activation protein (DAP12), and it activates TLR and NLR signaling pathways via secretion of pro-inflammatory cytokines and chemokines in response to microbial infection^57^. TREM-1 signaling has been indicated to play role in the pathogenesis of several inflammatory disorders of both infectious and non-infectious etiology. However, the significance of TREM signaling in innate immunity to virus infections and the underlying mechanisms remain largely unclear. Our data demonstrates that activation of TREM1 signaling is specific to pathogenic WNV NY99 infection. Majority of the genes involved in TREM-1 pathway including TLR, NLR, MCP-1, JAK2, IL-1β, cell adhesion and co-stimulator molecules, were up-regulated after WNV NY99 infection (Fig. 6A). In contrast, only MCP-1 and NLRP6 were up-regulated after WNV Eg101 infection (Fig. 6B). This finding could partially explain the high inflammatory response observed following WNV NY99 infection, as TREM-1 is directly implicated in the enhancement of inflammation by activating TLR and NLR signaling pathways. A substantial amount of data supports the link between TREM-1 signaling and several diseases, such as polymicrobial septic shock and inflammatory bowel disease, and in animal models of pneumonia and asthma^57^. TREM-1 signaling contributes to the cytokine storm associated with lethal filovirus disease^58^,^59^. It has been recently demonstrated that TREM-1 deficient mice were protected from severe influenza disease^60^. Another recent study by Suthar *et al*. used transcriptional profiling and pathway modeling to show that TREM-1 signaling was enriched in the liver following infection with WNV in mice^61^. Although this study implies that TREM-1 signaling might be one of the pathways responsible for restricting tissue tropism, the precise role of TREM-1 in WNV pathogenesis has not been explored. Based on our data, it is likely that TREM-1 signaling may promote inflammation and cause substantial tissue damage thereby contributing to disease pathogenesis of WNV NY99 infection.

### Death receptor signaling

**Figure 7:**
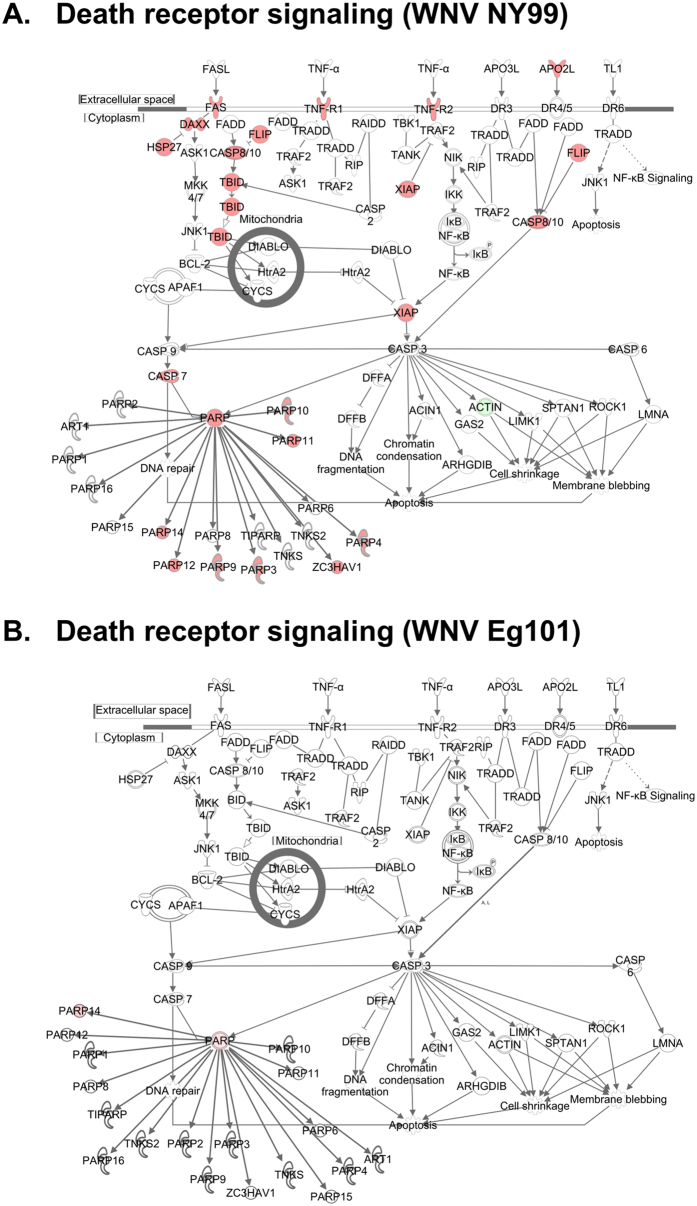
Pathway analysis for death receptor signaling. Genes associated with death receptor signaling activated by (**A**) WNV NY99 and (**B**) WNV Eg101 are shown. Differentially expressed genes are highlighted in color. Color intensity indicates the degree of up-regulation (red) or down-regulation (green) relative to the mock-infected mice brains. Solid lines represent direct interactions and dashed lines indirect interactions.

Many viruses encode proteins that can inhibit apoptosis and the triggering of apoptosis has been noted in WNV NY99-infected neuronal tissue^62^. We next generated a network map of death receptor signaling for WNV NY99- and WNV Eg101-infected mice. FasL, TNF-α, and Apo2L (TRAIL)-mediated downstream signaling leading to cell apoptosis were only activated in WNV NY99-infected mice, which also correlate with more severe pathogenicity observed in WNV NY99-infected mice (Fig. 7A,B). Downstream genes involved in cell death (DAXX, FLIP, TBID, PARP, CASP7, and CASP8) were activated upon WNV NY99 infection, but not following WNV Eg101 infection.

*In Summary, w*e have provided a comprehensive transcriptome analysis of mice brains that were infected with lethal and non-lethal WNV strains. One of the limitations of this study is the lack of over-expression or knockdown studies to delineate the role of the genes involved in the observed signaling pathways. Differential expression of specific host genes observed between virulent and non-virulent WNV strains could explain the variation in disease pathogenicity. In addition, we have identified novel genes and pathways that are not previously associated with WNV infection and might provide novel therapeutic targets for the management of WNVE.

## Materials and Methods

### Animal experiments

Nine-week old C57BL/6J mice were purchased from The Jackson Laboratory. Animals were housed four per cage and allowed to eat and drink *ad libitum*. The animal suite was maintained at 72 °F, 45% humidity and on 12/12-light/dark cycles. Sawdust bedding was provided along with paper towel. This study was approved by the University of Hawaii Institutional Animal Care and Use Committee (IACUC) (protocol number 13–1759), and conducted in strict accordance with guidelines established by the National Institutes of Health and the University of Hawaii IACUC. All animal experiments were conducted in consultation with veterinary and animal care staff at the University of Hawaii in the animal biosafety level-3 laboratory.

Mice (four animals per group) were inoculated via the footpad route with 100 plaque-forming units (PFU) of WNV NY99 or WNV Eg101 or PBS (mock). A power calculation assuming a type I error of 0.05 gave a power of 92% using four mice per group. At day 8 after infection, mice were anesthetized using isoflurane and perfused with cold PBS as described previously^10^. Brains were harvested and flash frozen in 2-methylbutane (Sigma) and stored at −80 °C until further use.

### Plaque assay

One-half of the frozen brain was weighed and homogenized in a bullet blender (Next Advance) using glass or zirconium beads, and plaque assay was conducted using Vero cells for analysis of WNV titer as described previously^10^,^63^.

### Gene expression analysis using RNA-seq

One-half of the frozen brain from four mice was powdered over dry ice to obtain a homogenous sampling. An aliquot of the frozen powder was used to isolate total RNA using RNeasy Mini Kit (Qiagen, Cat #217004) as described previously^63^. Genomic DNA contamination was eliminated by digesting the RNA with RNase-free DNase (Ambion, Austin, TX). The quality of all RNA samples was assessed using an Agilent Bioanalyzer 2100 (Agilent Technologies, Santa Clara, CA, USA). RNA-seq libraries were constructed using the Illumina TruSeq RNA sample preparation v2 as per manufacturer’s protocol (Illumina, San Diego, CA, USA), to generate 100-bp paired-end reads. Libraries were sequenced using an Illumina HiSeq 2500 with v3 chemistry at the University of California Irvine’s Genomic High Throughput facility. A total of 1,227,480,292 reads were inspected using the FastQC (http://www.bioinformatics.babraham.ac.uk/projects/fastqc/) program and minimally trimmed to remove low quality bases. The processed reads were then mapped to the mouse genome and the its gene annotations (mm_ref_GRCm38.p3) using TopHat (ver. 2.0.9), allowing for two mismatches^64^. The Cufflinks program (version 2.2) was used with the multi-read-correct option to estimate the normalized transcription profiles from the mapping results^65^. The Cuffdiff program from Cufflinks was used with a Benjamini-Hochberg False Discovery Rate (FDR) cut-off of 5% to assess statistically significant differential expression changes between the control and each of the infected groups.

### Pathway analysis

Data were analyzed using IPA (QIAGEN Redwood City, USA) as described previously^29^. The most significant canonical pathways and functional processes of biological importance were selected using the list of differentially expressed genes identified by RNA-seq and the IPA Knowledge Base as described previously^8^,^9^. Pathway enrichment p-values (Fisher’s exact test) and activation z-scores were calculated by IPA. Fisher’s exact test, using IPA, was used to calculate cut-off point of significance. p < 0.05 is considered significant.

### Validation by qRT-PCR

qRT-PCR was conducted on additional virus-infected (n = 7 in each group) and mock-infected (n = 4) brains. Brain tissues were powdered over dry ice to obtain a homogenous sampling and an aliquot of the frozen brain powder was used to extract total RNA as described previously. The mRNA levels of multiple host genes were determined using qRT-PCR and the fold-change in infected brains compared to mock brains was calculated after normalizing to the β-actin gene as described previously^63^. The primer sequences used for qRT-PCR are listed in Table 6.

### Statistical Analysis

Differentially expressed genes with an FDR adjusted p-value of the cuffdiff test statistics <0.05 were considered significant. For plaque assay and qRT-PCR, unpaired student t-test using Graphpad was used to calculate p values as described previously^10^,^66^. p < 0.05 is considered significant.

## Additional Information

**How to cite this article**: Kumar, M. *et al*. Identification of host genes leading to West Nile virus encephalitis in mice brain using RNA-seq analysis. *Sci. Rep*. **6**, 26350; doi: 10.1038/srep26350 (2016).

## Supplementary Material

Supplementary Table S1

Supplementary Table S2

## Figures and Tables

**Table 1:** Top 10 up-regulated and down-regulated differentially expressed genes in WNV NY99-infected mice brains.

Gene symbol	Gene name	Log_2_FC	p-value	FDR
CXCL10	C-X-C motif chemokine 10	9.6	5.00E-05	0.00159
SLFN4	Schlafen 4	9.3	5.00E-05	0.00159
MX1	Interferon-induced GTP-binding protein	8.7	5.00E-05	0.00159
LCN2	Lipocalin 2	8.3	5.00E-05	0.00159
CCL5	Chemokine (C-C Motif) Ligand 5	8.0	5.00E-05	0.02225
OASL1	2′-5′ oligoadenylate synthetase-like 1	7.7	5.00E-05	0.00159
CCL2	Chemokine (C-C Motif) Ligand 2	7.6	5.00E-05	0.00159
PYDC4	Pyrin domain containing 4	7.6	5.00E-05	0.00159
9330175E14Rik	RIKEN cDNA 9330175E14 gene	7.5	0.00025	0.00661
Ly6c2	Lymphocyte antigen 6 complex, locus C2	7.5	5.00E-05	0.00159
XIST	X-inactive specific transcript	−9.6	5.00E-05	0.00159
GKN3	Gastrokine 3	−4.6	5.00E-05	0.00159
MPZ	Myelin protein zero	−3.4	5.00E-05	0.00159
FAM65A	Family with sequence similarity 65, member A	−2.8	5.00E-05	0.00159
DANCR	Differentiation antagonizing non-protein coding RNA	−2.7	5.00E-05	0.00159
FZD10	Frizzled class receptor 10	−2.7	0.00045	0.01091
EPX	Eosinophil peroxidase	−2.6	0.00025	0.00661
SPON2	Spondin 2	−2.4	0.00105	0.02225
CAPN11	Calpain 11	−2.3	0.00015	0.00423
PF4	Platelet factor 4	−2.3	0.0004	0.00986

**Table 2:** Top 10 up-regulated and down-regulated differentially expressed genes in WNV Eg101-infected mice brains.

Gene symbol	Gene name	Log_2_FC	p-value	FDR
GH	Growth hormone	7.4	5.00E-05	0.00159
PRL	Prolactin	7.2	5.00E-05	0.00159
CGA	Glycoprotein hormones	6.6	5.00E-05	0.00159
PYDC4	Pyrin domain containing 4	3.2	5.00E-05	0.00159
MX1	Interferon-induced GTP-binding protein	3.0	5.00E-05	0.02225
OASL2	2′-5′ oligoadenylate synthetase-like 2	2.7	5.00E-05	0.00159
IFI44	Interferon-induced protein 44	2.7	5.00E-05	0.00159
POMC	Proopiomelanocortin	2.6	5.00E-05	0.00159
USP18	Ubiquitin specific peptidase 18	2.6	5.00E-05	0.00159
PITX1	Paired-like homeodomain 1	2.4	0.00225	0.04115
DANCR	Differentiation antagonizing non-protein coding RNA	−2.6	5.00E-05	0.00159
FAM65A	Family with sequence similarity 65, member A	−2.6	5.00E-05	0.00159
POU4F1	POU Class 4 Homeobox 1	−2.4	5.00E-05	0.00159
–	Unknown	−2.4	0.0018	0.03461
HOXB5	Homeobox B5	−2.2	5.00E-05	0.00159
–	Unknown	−2.2	5.00E-05	0.00159
HOXD1	Homeobox D1	−2.0	0.0023	0.04179
ALPNR	Apelin receptor	−1.7	0.00035	0.00879
H19	H19	−1.7	0.0004	0.00986
CNPY1	Canopy FGF signaling regulator 1	−1.6	5.00E-05	0.00159

**Table 3:** Commonly expressed genes demonstrating inverse trend in WNV NY99 and WNV Eg101 infections.

Gene symbol	Gene name	Log_2_FC (WNV NY99)	Log_2_FC (WNV Eg101)
HOXB5	Homeobox B5	1.4	−2.2
GFAP	Glial fibrillary acidic protein	1.3	−0.5
HBA-A1	Hemoglobin alpha, adult chain 1	1.0	−1.1
CYR61	Cysteine-rich angiogenic inducer 61	0.8	−1.0
α2M	Alpha-2-macroglobulin	0.6	−0.6
SYNPO2	Synaptopodin 2	−1.5	0.7
PTPN3	Protein tyrosine phosphatase, non-receptor type 3	−0.6	0.6

**Table 4:** Gene enrichment in interferon signaling and inflammatory response.

Interferon response			Inflammatory mediators		
Gene symbol	Log_2_FC (WNV NY99)	Log_2_FC (WNV Eg101)	Gene symbol	Log_2_FC (WNV NY99)	Log_2_FC (WNV Eg101)
MX1	8.7	3.0	CXCL10	9.7	NC*
OASL1	7.7	2.1	CCL5	8.0	NC
IFI44	7.4	2.7	CCL2	7.9	NC
RSAD2	7.2	2.0	CCL7	6.6	NC
OASL2	7.1	2.7	CXCL1	6.2	NC
IFIT1	7.0	2.3	CXCL12	5.7	1.9
OAS1b	6.7	2.0	CXCL13	5.3	NC
IFIT3	6.7	2.0	CCL4	5.1	NC
IFI204	6.6	2.3	CCL3	5.7	NC
PYHIN1	6.5	2.1	CCL8	4.1	NC
OAS1a	6.5	1.5	IL-1β	3.7	NC
IFIT3b	6.3	1.9	IL7	2.9	NC
OAS2	6.0	2.0	CCL19	2.6	NC
MX2	5.9	1.5	CXCL16	2.5	NC
IFIT2	5.8	1.2	IL-1α	2.2	NC

*No Change.

**Table 5:** Expression of toll-like receptors.

Gene symbol	Log_2_FC (WNV NY99)	Log_2_FC (WNV Eg101)
TLR2	3.4	NC*
TLR3	2.8	NC
TLR4	2.7	NC
TLR9	2.2	NC
TLR1	2.1	NC
TLR7	2.0	NC
TLR13	1.8	NC
TLR8	1.0	NC

*No Change.

**Table 6:** Primer sequences used for qRT-PCR.

Gene (Accession No.)	Primer Sequence (5′-3′)
α2M (NM_175628)	
Forward	CTACAAGGTGGTGATACG
Reverse	CGGACACATTCATCTCTT
MX1 (NM_010846)	
Forward	CTCATTAGCCTGGATGTC
Reverse	ATGTATGTCTTGATAAGTCTCT
IL-1β (NM_008361)	
Forward	CCTCACAAGCAGAGCACAAG
Reverse	AAACAGTCCAGCCCATACTTTAG
IFIT1 (NM_008331)	
Forward	GTTGTTGTTGTTGTTCGT
Reverse	CAGCAGGAATCAGTTGTG
GFAP (NM_010277)	
Forward	GTGGATTTGGAGAGAAAG
Reverse	GTATTGAGTGCGAATCTC
IFIT3 (NM_010501)	
Forward	GTCCTCTCTACTCTTTGG
Reverse	CATCCTCTGTCTTCTCTC
CCL3 (NM_011337)	
Forward	ATTCCACGCCAATTCATC
Reverse	ATTCAGTTCCAGGTCAGT

## References

[b1] Hayes E. B. & Gubler D. J. West Nile virus: epidemiology and clinical features of an emerging epidemic in the United States. Annu Rev Med 57, 181–194 (2006). 16409144 10.1146/annurev.med.57.121304.131418

[b2] Donadieu E. . Differential virulence and pathogenesis of West Nile viruses. Viruses 5, 2856–2880 (2013). 24284878 10.3390/v5112856 PMC3856419

[b3] Lanciotti R. S. . Complete genome sequences and phylogenetic analysis of West Nile virus strains isolated from the United States, Europe, and the Middle East. Virology 298, 96–105 (2002). 12093177 10.1006/viro.2002.1449

[b4] Calistri P. . Epidemiology of west nile in europe and in the mediterranean basin. O Virol J 4, 29–37 (2010). 10.2174/1874357901004010029 PMC2878979 20517490

[b5] Melnick J. L. . Isolation from human sera in Egypt of a virus apparently identical to West Nile virus. Exp Biol Med 77, 661–665 (1951). 10.3181/00379727-77-18884 14891830

[b6] Kumar M., O’Connell M., Namekar M. & Nerurkar V. R. Infection with non-lethal West Nile virus Eg101 strain induces immunity that protects mice against the lethal West Nile virus NY99 strain. Viruses 6, 2328–2339 (2014). 24915459 10.3390/v6062328 PMC4074930

[b7] Kumar M., Roe K., O’Connell M. & Nerurkar V. R. Induction of virus-specific effector immune cell response limits virus replication and severe disease in mice infected with non-lethal West Nile virus Eg101 strain. J Neuroinflammation 12, 178 (2015). 26392176 10.1186/s12974-015-0400-y PMC4578235

[b8] Kim J. A., Park S. K., Kumar M., Lee C. H. & Shin O. S. Insights into the role of immunosenescence during varicella zoster virus infection (shingles) in the aging cell model. Oncotarget 6, 35324–35343 (2015). 26473290 10.18632/oncotarget.6117 PMC4742108

[b9] Park S. J. . Dynamic changes in host gene expression associated with H5N8 avian influenza virus infection in mice. Sci Rep 5, 16512 (2015). 26576844 10.1038/srep16512 PMC4649622

[b10] Kumar M. . Impaired virus clearance, compromised immune response and increased mortality in type 2 diabetic mice infected with West Nile virus. PLoS One 7, e44682 (2012). 22953001 10.1371/journal.pone.0044682 PMC3432127

[b11] Roe K. . West Nile virus-induced disruption of the blood-brain barrier in mice is characterized by the degradation of the junctional complex proteins and increase in multiple matrix metalloproteinases. J Gen Virol 93, 1193–203 (2012). 22398316 10.1099/vir.0.040899-0 PMC3755517

[b12] Verma S., Kumar M. & Nerurkar V. R. Cyclooxygenase-2 inhibitor blocks the production of West Nile virus-induced neuroinflammatory markers in astrocytes. J Gen Virol 92, 507–515 (2011). 21106803 10.1099/vir.0.026716-0 PMC3081232

[b13] van Marle G. . West Nile virus-induced neuroinflammation: glial infection and capsid protein-mediated neurovirulence. J Virol 81, 10933–10949 (2007). 17670819 10.1128/JVI.02422-06 PMC2045515

[b14] Moestrup S. K., Gliemann J. & Pallesen G. Distribution of the alpha 2-macroglobulin receptor/low density lipoprotein receptor-related protein in human tissues. Cell Tissue Res 269, 375–382 (1992). 1423505 10.1007/BF00353892

[b15] Gourine A. V. . Role of alpha(2)-macroglobulin in fever and cytokine responses induced by lipopolysaccharide in mice. Am J Physiol Regul Integr Comp Physiol 283, R218–226 (2002). 12069948 10.1152/ajpregu.00746.2001

[b16] Umans L. . Targeted inactivation of the mouse alpha 2-macroglobulin gene. J Biol Chem 270, 19778–19785 (1995). 7544347 10.1074/jbc.270.34.19778

[b17] Chen H., Li Z., Liu N., Zhang W. & Zhu G. Influence of Alpha-2-Macroglobulin 5 bp I/D and Ile1000Val polymorphisms on the susceptibility of Alzheimer’s disease: a systematic review and meta-analysis of 52 studies. Cell Biochem Biophys 70, 511–519 (2014). 24756728 10.1007/s12013-014-9950-3

[b18] Diamond M. S. & Gale M. Jr. Cell-intrinsic innate immune control of West Nile virus infection. Trends Immunol 33, 522–530 (2012). 22726607 10.1016/j.it.2012.05.008 PMC3461102

[b19] Szretter K. J. . The interferon-inducible gene viperin restricts West Nile virus pathogenesis. J Virol 85, 11557–11566 (2011). 21880757 10.1128/JVI.05519-11 PMC3209274

[b20] Cho H., Shrestha B., Sen G. C. & Diamond M. S. A role for Ifit2 in restricting West Nile virus infection in the brain. J Virol 87, 8363–8371 (2013). 23740986 10.1128/JVI.01097-13 PMC3719802

[b21] Samuel M. A. . PKR and RNase L contribute to protection against lethal West Nile Virus infection by controlling early viral spread in the periphery and replication in neurons. J Virol 80, 7009–7019 (2006). 16809306 10.1128/JVI.00489-06 PMC1489062

[b22] Scherbik S. V., Paranjape J. M., Stockman B. M., Silverman R. H. & Brinton M. A. RNase L plays a role in the antiviral response to West Nile virus. J Virol 80, 2987–2999 (2006). 16501108 10.1128/JVI.80.6.2987-2999.2006 PMC1395436

[b23] Lim J. K. . Genetic variation in OAS1 is a risk factor for initial infection with West Nile virus in man. PLoS Pathog 5, e1000321 (2009). 19247438 10.1371/journal.ppat.1000321 PMC2642680

[b24] Klein R. S. . Neuronal CXCL10 directs CD8^+^ T-cell recruitment and control of West Nile virus encephalitis. J Virol 79, 11457–11466 (2005). 16103196 10.1128/JVI.79.17.11457-11466.2005 PMC1193600

[b25] Glass W. G. . Chemokine receptor CCR5 promotes leukocyte trafficking to the brain and survival in West Nile virus infection. J Exp Med 202, 1087–1098 (2005). 16230476 10.1084/jem.20042530 PMC2213214

[b26] Getts D. R. . Ly6c+ “inflammatory monocytes” are microglial precursors recruited in a pathogenic manner in West Nile virus encephalitis. J Exp Med 205, 2319–2337 (2008). 18779347 10.1084/jem.20080421 PMC2556789

[b27] Durrant D. M., Robinette M. L. & Klein R. S. IL-1R1 is required for dendritic cell-mediated T cell reactivation within the CNS during West Nile virus encephalitis. J Exp Med 210, 503–516 (2013). 23460727 10.1084/jem.20121897 PMC3600909

[b28] Qian F. . Identification of genes critical for resistance to infection by West Nile virus using RNA-seq analysis. Viruses 5, 1664–1681 (2013). 23881275 10.3390/v5071664 PMC3738954

[b29] Clarke P., Leser J. S., Bowen R. A. & Tyler K. L. Virus-induced transcriptional changes in the brain include the differential expression of genes associated with interferon, apoptosis, interleukin 17 receptor A, and glutamate signaling as well as flavivirus-specific upregulation of tRNA synthetases. MBio 5, e00902–00914 (2014). 24618253 10.1128/mBio.00902-14 PMC3952157

[b30] Gupta N. & Rao P. V. Transcriptomic profile of host response in Japanese encephalitis virus infection. Virol J 8, 92 (2011). 21371334 10.1186/1743-422X-8-92 PMC3058095

[b31] Yang Y. . Japanese encephalitis virus infection induces changes of mRNA profile of mouse spleen and brain. Virol J 8, 80 (2011). 21345237 10.1186/1743-422X-8-80 PMC3056812

[b32] Kumar M. & Nerurkar V. R. Integrated analysis of microRNAs and their disease related targets in the brain of mice infected with West Nile virus. Virology 452–453, 143–151 (2014). 10.1016/j.virol.2014.01.004 PMC3959158 24606691

[b33] Shin O. S., Kumar M., Yanagihara R. & Song J. W. Hantaviruses induce cell type- and viral species-specific host microRNA expression signatures. Virology 446, 217–224 (2013). 24074584 10.1016/j.virol.2013.07.036 PMC4129941

[b34] Shin O. S. . Hantaviruses induce antiviral and pro-inflammatory innate immune responses in astrocytic cells and the brain. Viral Immunol 27, 256–266 (2014). 24937036 10.1089/vim.2014.0019 PMC4076983

[b35] Janeway C. A. Jr. & Medzhitov R. Innate immune recognition. Annu Rev Immunol 20, 197–216 (2002). 11861602 10.1146/annurev.immunol.20.083001.084359

[b36] Fredericksen B. L. The neuroimmune response to West Nile virus. J Neurovirol 20, 113–121 (2014). 23843081 10.1007/s13365-013-0180-z PMC3971464

[b37] Suthar M. S., Diamond M. S. & Gale M. Jr. West Nile virus infection and immunity. Nat Rev Microbiol 11, 115–128 (2013). 23321534 10.1038/nrmicro2950

[b38] Fredericksen B. L., Keller B. C., Fornek J., Katze M. G. & Gale M. Jr. Establishment and maintenance of the innate antiviral response to West Nile Virus involves both RIG-I and MDA5 signaling through IPS-1. J Virol 82, 609–616 (2008). 17977974 10.1128/JVI.01305-07 PMC2224571

[b39] Suthar M. S. . IPS-1 is essential for the control of West Nile virus infection and immunity. PLoS Pathog 6 **(2)**, e1000757 (2010). 20140199 10.1371/journal.ppat.1000757 PMC2816698

[b40] Errett J. S., Suthar M. S., McMillan A., Diamond M. S. & Gale M. Jr. The essential, nonredundant roles of RIG-I and MDA5 in detecting and controlling West Nile virus infection. J Virol 87, 11416–11425 (2013). 23966395 10.1128/JVI.01488-13 PMC3807316

[b41] Lazear H. M. . Pattern recognition receptor MDA5 modulates CD8^+^ T cell-dependent clearance of West Nile virus from the central nervous system. J Virol 87 **(21)**, 11401–11415 (2013). 23966390 10.1128/JVI.01403-13 PMC3807324

[b42] Akira S., Uematsu S. & Takeuchi O. Pathogen recognition and innate immunity. Cell 124, 783–801 (2006). 16497588 10.1016/j.cell.2006.02.015

[b43] Wang T. . Toll-like receptor 3 mediates West Nile virus entry into the brain causing lethal encephalitis. Nat Med 10, 1366–1373 (2004). 15558055 10.1038/nm1140

[b44] Daffis S., Samuel M. A., Suthar M. S., Gale M. Jr. & Diamond M. S. Toll-like receptor 3 has a protective role against West Nile virus infection. J Virol 82, 10349–10358 (2008). 18715906 10.1128/JVI.00935-08 PMC2573187

[b45] Welte T. . Toll-like receptor 7-induced immune response to cutaneous West Nile virus infection. J Gen Virol 90, 2660–2668 (2009). 19641044 10.1099/vir.0.011783-0 PMC2771433

[b46] Town T. . Toll-like receptor 7 mitigates lethal West Nile encephalitis via interleukin 23-dependent immune cell infiltration and homing. Immunity 30, 242–253 (2009). 19200759 10.1016/j.immuni.2008.11.012 PMC2707901

[b47] Hines D. J., Choi H. B., Hines R. M., Phillips A. G. & MacVicar B. A. Prevention of LPS-induced microglia activation, cytokine production and sickness behavior with TLR4 receptor interfering peptides. PLoS One 8, e60388 (2013). 23555964 10.1371/journal.pone.0060388 PMC3610686

[b48] Baenziger S. . Triggering TLR7 in mice induces immune activation and lymphoid system disruption, resembling HIV-mediated pathology. Blood 113, 377–388 (2009). 18824599 10.1182/blood-2008-04-151712

[b49] Marcondes M. C., Spina C., Bustamante E. & Fox H. Increased toll-like receptor signaling pathways characterize CD8^+^ cells in rapidly progressive SIV infection. Biomed Res Int 2013, 796014 (2013). 23484159 10.1155/2013/796014 PMC3591242

[b50] Kong K. F. . Dysregulation of TLR3 impairs the innate immune response to West Nile virus in the elderly. J Virol 82, 7613–7623 (2008). 18508883 10.1128/JVI.00618-08 PMC2493309

[b51] Sejvar J. J., Lindsey N. P. & Campbell G. L. Primary causes of death in reported cases of fatal West Nile Fever, United States, 2002–2006. Vector Borne Zoonotic Dis 11, 161–164 (2011). 20687860 10.1089/vbz.2009.0086

[b52] Shirey K. A. . The TLR4 antagonist Eritoran protects mice from lethal influenza infection. Nature 497, 498–502 (2013). 23636320 10.1038/nature12118 PMC3725830

[b53] Sabouri A. H. . TLR signaling controls lethal encephalitis in WNV-infected brain. Brain Res 1574, 84–95 (2014). 24928618 10.1016/j.brainres.2014.05.049 PMC4099315

[b54] Ramos H. J. . IL-1beta signaling promotes CNS-intrinsic immune control of West Nile virus infection. PLoS Pathog 8, e1003039 (2012). 23209411 10.1371/journal.ppat.1003039 PMC3510243

[b55] Kaushik D. K., Gupta M., Kumawat K. L. & Basu A. NLRP3 Inflammasome: Key mediator of neuroinflammation in murine Japanese encephalitis. PLoS One 7(**2**), e32270 (2012). 22393394 10.1371/journal.pone.0032270 PMC3290554

[b56] Kumar M. . Inflammasome adaptor protein Apoptosis-associated speck-like protein containing CARD (ASC) is critical for the immune response and survival in West Nile virus encephalitis. J Virol 87, 3655–3667 (2013). 23302887 10.1128/JVI.02667-12 PMC3624239

[b57] Roe K., Gibot S. & Verma S. Triggering receptor expressed on myeloid cells-1 (TREM-1): a new player in antiviral immunity? Front Microbiol 5, 627 (2014). 25505454 10.3389/fmicb.2014.00627 PMC4244588

[b58] Mohamadzadeh M. . Activation of triggering receptor expressed on myeloid cells-1 on human neutrophils by marburg and ebola viruses. J Virol 80, 7235–7244 (2006). 16809329 10.1128/JVI.00543-06 PMC1489070

[b59] Wauquier N., Becquart P., Padilla C., Baize S. & Leroy E. M. Human fatal zaire ebola virus infection is associated with an aberrant innate immunity and with massive lymphocyte apoptosis. PLoS Negl Trop Dis 4, e837 (2010). 20957152 10.1371/journal.pntd.0000837 PMC2950153

[b60] Weber B. . TREM-1 deficiency can attenuate disease severity without affecting pathogen clearance. PLoS Pathog 10, e1003900 (2014). 24453980 10.1371/journal.ppat.1003900 PMC3894224

[b61] Suthar M. S. . A systems biology approach reveals that tissue tropism to West Nile virus is regulated by antiviral genes and innate immune cellular processes. PLoS Pathog 9, e1003168 (2013). 23544010 10.1371/journal.ppat.1003168 PMC3567171

[b62] Shrestha B., Gottlieb D. & Diamond M. S. Infection and injury of neurons by West Nile encephalitis virus. J Virol 77, 13203–13213 (2003). 14645577 10.1128/JVI.77.24.13203-13213.2003 PMC296085

[b63] Kumar M. . Reduced immune cell infiltration and increased pro-inflammatory mediators in the brain of Type 2 diabetic mouse model infected with West Nile virus. J Neuroinflammation 11, 80 (2014). 24750819 10.1186/1742-2094-11-80 PMC4001407

[b64] Trapnell C., Pachter L. & Salzberg S. L. TopHat: discovering splice junctions with RNA-seq. Bioinformatics 25, 1105–1111 (2009). 19289445 10.1093/bioinformatics/btp120 PMC2672628

[b65] Trapnell C. . Differential gene and transcript expression analysis of RNA-seq experiments with TopHat and Cufflinks. Nat Protoc 7, 562–578 (2012). 22383036 10.1038/nprot.2012.016 PMC3334321

[b66] Kumar M., Verma S. & Nerurkar V. R. Pro-inflammatory cytokines derived from West Nile virus (WNV)-infected SK-N-SH cells mediate neuroinflammatory markers and neuronal death. J Neuroinflammation 7, 73 (2010). 21034511 10.1186/1742-2094-7-73 PMC2984415

